# Exploring the adoption of concept-based curricula: insights from educators and implications for change

**DOI:** 10.1007/s10459-024-10346-y

**Published:** 2024-06-03

**Authors:** Judith Tweedie, Fiona Pelly, Hattie Wright, Claire Palermo

**Affiliations:** 1 Nutrition and Dietetics, School of Health, University of the Sunshine Coast, Sippy Downs, QLD Australia; 2 Office of the Deputy Dean Education, Faculty of Medicine, Nursing and Health Sciences, Monash University, Clayton, VIC Australia

**Keywords:** Dietetics, Competency-based education, Diffusion of innovation

## Abstract

Concept-based approaches to curriculum design have been proposed to solve content and curricula overload and promote conceptual learning. Few health professions have adopted this approach and little is known about how to support this educational change. We aimed to understand how nutrition and dietetics educators may navigate proposed education change towards concept-based curricula. We employed an interpretivist approach and in-depth interviews that explored the views of nutrition and dietetic educators towards using a concept-based approach to curriculum. Employing deductive thematic analysis based on the diffusion of innovation theory, data from twenty experienced dietetics educators were analysed. Three main themes were identified; the need for change champions, concerns about change, and the complexity of the education system. Diffusion of innovation theory highlighted that to enact change, the relative advantage and compatibility of the approach with current structures and systems, with evidence from trialling and observing the new approach in action, were needed. Developing education leaders and infiltrating the social system of education through existing communities of practice is critical to enacting educational change.

## Introduction

To meet the pace of change occurring in healthcare, globalization, and rapid advancements in health technology, health professions education must undergo continual change. This constant need for change, and associated content, has led to an overcrowded curricula, placing burden on faculty to find “space” for such content and stress on students to “know more”. Traditional approaches to health professional education have been described as ‘two-dimensional’, where learning is focused on facts and skills, and can result in superficial coverage of content and cognitive overload in learners, leading to a lack of deep understanding and critical thinking (D’Eon, 2023; Erickson et al., 2017; Giddens & Brady, 2007). An overly crowded curriculum has been recognized as a challenge to reform in health professional education (D’Eon, 2023; Institute of Medicine, 2003). Some have argued that this crowded curriculum constrains the scholarly capacity of students, diminishing curiosity, leading to poor student health outcomes (D’Eon, 2023). Concept-based approaches to curriculum design have been described as three-dimensional, organised around concepts not content, and proposed to solve content overload, crowding and promote conceptual learning. Concepts are mental constructs of one- or two-word nouns or short phrases that are timeless, universal, abstract and exhibit a higher level of abstraction than content topics (Erickson et al., 2017; Rowland et al., 2011). Concepts are derived from the knowledge and processes defined by a discipline (Erickson et al., 2017), and describe and construct behaviors and meanings (Hardin & Richardson, 2012). Concepts replace content or topics in curricula, and allow for current exemplars to be used to illustrate application of the concept. The process for developing a concept-based curriculum starts with identification of the core (foundational) concepts of the discipline and their definitions (Giddens & Brady, 2007). Concept-based curricula have been adopted in nursing across the United states (Brussow et al., 2019) and New Zealand (McGrath, 2015) and in pharmacology (Guilding et al., 2023), yet little is known about the transferability to other health professions.

A concept-based curriculum draws on the principle of conceptual learning. Conceptual learning has been defined as being able to recognize patterns and linkages within information, and being able to see the relevance of the knowledge to different situations (Fletcher et al., 2019). Theorist have argued that conceptual learning supports deeper understandings, and that learners benefit from this as they can solve unfamiliar problems, not just those they have previously been exposed to (Maclellan, 2005). This involves “symbolic manipulations in the head” of the learner whereby they can extract aspects of knowledge and draw relevant parts together and apply to different situations (Giddens & Brady, 2007; Maclellan, 2005). The experiential learning through practical placements that are a foundation of all health professions education, presents learners with patient conditions and situations that may have not been presented to them in classroom base curriculum. Conceptual learning postulates that students will be able to identify aspects of the condition and know what to do although the condition is unfamiliar (Giddens & Brady, 2007). This form of learning is therefore highly advantageous as health system needs and patient cases increase in complexity.

Innovations in higher education, such as concept-based curricula can be either ‘disruptive’ or ‘sustainable’ (Tierney & Lanford, 2016). Change is defined as a “transitional process with multiple and varied events supporting the objective of moving an organisation and its stakeholders from a current state to a future state” (Association of Change Management Professionals (ACMP), 2019)^p.8^ As with any change process, curriculum reform can be challenging. Resistance and difficulties in steering the change process are the main challenges faced by academics in medical education when driving significant curriculum reform (Velthuis et al., 2018). The curriculum orientation of a discipline has also been found to influence and shape academics curriculum decisions and the way they respond to educational changes (Roberts, 2015). Successful change management requires an understanding of the institutional culture and the individual’s unique perspectives and beliefs which either impede or increase acceptance and commitment to change (Association of Change Management Professionals (ACMP), 2019). Insights into these perspectives and beliefs are critical for instigating change in health professions education.

While the challenges of change in health professions education have been described (Sundberg et al., 2017; Velthuis et al., 2018), these studies do not draw on change theory to explain the process of change or draw from this theory to guide change efforts. Activity theory has been used to better understand how stakeholders navigate change and support them through reform (Law et al., 2022). In addition, the role of leadership has also been explored in regards to curriculum reform noting the requirement of leaders to navigate multiple stakeholders and manage resistance to change (Velthuis et al., 2018). While institutional culture and an individual’s unique beliefs and values are known to influence the change process (Association of Change Management Professionals (ACMP), 2019), Rogers’ diffusion of innovation theory may also assist understanding of change in health professions education. Diffusion of innovation theory describes how people within a system learn and make decisions about a new or innovative practice. The theory espouses five stages (1) knowledge and awareness of new innovative practices, (2) persuasion—gaining support for the innovative practice, (3) decision—the intention to try the innovative practice, (4) implementation—trailing or delivering the innovative practice, and (5) confirmation—support to continue the innovative practice (Rogers, 2003). Within this theory, diffusion is the process by which change occurs such that the system within which the phenomenon exists is different to what it was before. It has been used to explore the adoption of evidence based practice in health services highlighting the importance of change agents and leadership (Greenhalgh et al., 2005) and to support change in medical education (Gonzalo et al., 2018). Using diffusion of innovation theory to help explain change in health professions education curriculum, warrants further exploration.

Dietetics is a small yet growing health profession with increasing recognition of their role beyond patient care. Studies internationally highlight the increasing demand for dietitians in community-based chronic disease prevention and management, aged care, personalized nutrition, food and agriculture, and digital health (Boak et al., 2022; Hickson et al., 2018; Kicklighter et al., 2017). Dietitians are health professionals who apply “the science of food and nutrition to promote health, prevent and treat disease to optimize the health of individuals, groups, communities and populations.” (International Confederation of Dietetic Associations, 2017). Across the Western world, dietetics education requires completion of a minimum of a Bachelor or undergraduate degree accredited by an independent regulatory council (or equivalent), with professional standards for maintaining professional competency and working within a code of conduct and to a scope of practice. Dietitians are recognized as a key part of the health care system internationally (Beckingsale et al., 2016; Jortberg & Fleming, 2014). The dietetics profession has recognized that the profession and its practice needs to evolve to be adaptable, work in new emerging areas of practice, and demonstrate critical thinking skills to deal with increasing medical complexity and an aging population (Boak et al., 2022; Hickson et al., 2018; Kicklighter et al., 2017). Internationally, data suggests that the dietetics curriculum needs to diversify, with a strong underpinning of science, to build in the curricula systems science, food, psychology and behavior change, innovation, social justice and equity, digital technologies, climate change and environmental sustainability, and advocacy concepts as well as the growing demands of an aging population (Boak et al., 2022; Kicklighter et al., 2017). Despite its relative youth, dietetics curricula suffers from overcrowding. To address this issue, fifty six core concepts were developed internationally based on existing and future competency standards across 10 countries (Tweedie et al., 2020) and confirmed by Australian dietitians (Tweedie et al., 2021). However, how these concepts can be successfully translated into dietetics curriculum is yet to be determined.

Our study aimed to understand how nutrition and dietetics educators may navigate proposed education change towards concept-based curriculum. More specifically it sought to answer two research questions:

1. What are the key factors influencing educational change in nutrition and dietetics education?
2. Can diffusion of innovation explain what would support or hinder curriculum reform to a concept-based approach to the education of future nutrition and dietetics professionals?

## Methods

### Study design

We employed an interpretivist approach as we sought to understand the multiple perspectives of nutrition and dietetic educators towards educational change to a concept-based approach to curriculum design and adopting core concepts. We then applied diffusion of innovation theory to explain what may be required to facilitate major education change. As dietetics educators (JT, FP, HW, CP) with experience in dietetics program accreditation (CP, FP), we recognised the unique position we had in the research and employed reflexivity through the data collection and analysis process whereby we challenged each other’s perspectives and interpretations as we sought meaning from the data. We employed individual semi-structured interviews to gain insights from Australian and New Zealand dietetic educators. The in-depth interviews aimed to explore the “insider perspective” of dietetic educators to capture their thoughts, feelings and perceptions of reforming curriculum using dietetic concepts, while at the same time providing opportunity for participants to provide novel perspectives (Liamputtong, 2017). While emerging evidence recognizes the important role students have in curriculum (Abbonizio et al., 2024), historically academic dietetics educators have been the drivers for curriculum and therefore they were the focus of this study. Ethics approval was obtained from the University of the Sunshine Coast Human Research Ethics Committee (approval number: S221686).

### Sampling

Our participants were selected using purposive and snowballing sampling (Liamputtong, 2017). Discipline leaders and program coordinators of the 18 (at the time of the study) accredited Australian (*n* = 15) and New Zealand (*n* = 3) universities with dietetics education programs. The participants were identified from the Council of Deans Nutrition and Dietetics (https://www.deansnutritiondietetics.com) and Nutrition and Dietetics Education and Accreditation Network (NaDEAN) who represent all Australian and New Zealand dietetics education programs. Snowball sampling was used to identify curriculum leaders within the programs. The concept of information power was employed to determine the sample size (Malterud et al., 2016). Given the specificity of the aim and potential sample together with the strong theoretical underpinning of change management, we aimed for a sample of approximately 20 participants. Participants were invited to participate via email in January 2022. Three follow-up recruitment emails were sent.

### Data collection

Interviews were conducted online between February and May 2022 via videoconference (Zoom video communications, Inc. California, 2023) to capture participants geographically dispersed across Australia and New Zealand. After initial interest was expressed, informed consent was obtained from participants before the interview. Interviews were informed by literature on the challenges of reforming curriculum (Velthuis et al., 2018) and change management processes (Association of Change Management Professionals (ACMP), 2019). Prior to the interview, participants were sent pre-reading on dietetic concepts and concept-based approaches to curriculum design. At the commencement of the interview, basic demographic questions were asked including: participants current position, location, years of experience as a dietitian and in dietetics education, and whether their university program was undergraduate or post graduate. Participants were initially asked what role they played in leading curriculum change within the dietetic education program. Further questions explored influences on changing curriculum and the challenges of changing curriculum to dietetic concepts (Table 1). After conducting each interview, one author (JT) completed a summary of initial impressions of the interview which was used to assist later in data analysis. Interviews were audio record and transcribed using Otter.ai automated transcription service and then corrected for accuracy. The ethics approval required the interview transcript to be de-identified and emailed to participants for verification and the opportunity to amend prior to analysis. Participants were asked to verify the transcript within two weeks of the transcript being emailed to them.

### Data analysis

We employed deductive thematic analysis to analyse the data. A coding framework was developed based on the diffusion of innovation theory encompassing five main codes reflecting the five stages of change to the innovation: relative advantage (the degree to which an innovation is perceived as being better than the existing alternatives); compatibility (the degree to which an innovation is perceived as consistent with the existing values, experiences, and needs of potential adopters); complexity (the degree to which an innovation is perceived as difficult to understand and use); trialability (the degree to which an innovation can be experimented with before making a decision to adopt it); and observability (the degree to which the results of an innovation can be observed by others) (Rogers, 2003). In addition, main codes included communication channels, time and social systems, as the three other key components to the diffusion process (Rogers, 2003) Table 2. Line by line coding of the data was undertaken by the last author against the coding framework with sub-codes added inductively and categorised under each of the main codes as coding progressed. Coding was then compared with initial impressions of the interview summaries for alignment of interpretation. Patterns across codes were then compared to research questions and drafted into themes to answer the two research questions. To support reflexivity, two other authors read and coded three different interview transcripts each to review the initial themes and descriptors to facilitate other potential interpretations. Three authors then came together to finalise themes and select illustrative quotes to support their presentation.

**Table 1:** Interview guide for dietetics curriculum agents guided by rogers diffusion of innovation theory(Rogers, 2003)

Question	Guiding theory
After examining the pre-reading material, what is your understanding of a concept - based approach to curriculum design?	Knowledge Relative advantage Complexity
If dietetic education programs were to adopt a concept-based approach what impact would this change have on your program? And why do you think this change would have this impact? Prompts: What do you think are the influences on this type of change occurring within your program? - prompt to ask what hinders or what supports change? What are the forces that shape your own decisions in implementing a concept-based approach to curriculum re-design?	Decision Compatibility Complexity Trialability Observability
From your perspective and the university’s, who are the external stakeholders and who are the internal stakeholders that need to be considered when changing dietetic curriculum? What is your perspective the role of the large groups of stakeholders influencing curriculum change? Prompts: What strategies would be required to mitigate resistance to changing dietetic curriculum to a concept-based curriculum? Why would these strategies work in your opinion? At the local (institutional) level? at the national level? What would be required to develop a shared vision between all the stakeholders to change curriculum to a concept-based approach?	Persuasion Decision Observability Trialability
What is your perspective on resistance to change as influencing curriculum change?	Persuasion Decision Observability Trialability
What would be required to steer curriculum change from traditional dietetic content to a concept-based approach to curriculum? Why? Prompts: Other potential barriers and enablers?	Persuasion Decision Implementation Observability Trialability
How do you think a concept-based approach to curriculum design could enable this diversification of dietetics curriculum to be more future focussed?	Implementation Maintenance Trialability

**Table 2:** Coding framework

The innovation Idea or practice that is new. i.e., concept-based curricula		
1.0 Relative Advantage	1.1 improved student outcomes 1.2 increased engagement 1.3 better preparation for the workforce 1.4 efficiencies in education	*The degree to which an innovation is perceived as being better than the existing alternatives i.e., the perceived benefits of changing dietetics education practice*
2.0 Compatibility	2.1 compatibility with existing curricula 2.2 teaching methodologies 2.3 pedagogical underpinning 2.4 student learning preferences 2.5 competing priorities 2.6 stakeholder perceptions/philosophies 2.7 stakeholder knowledge/capability 2.8 accreditation compatibility	*The degree to which an innovation is perceived as consistent with the existing values, experiences, and needs of potential adopters i.e., the degree to which changing dietetics education practice aligns with the values, goals, and needs of dietetics educators and students*
3.0 Complexity	3.1 level of effort required to make change 3.2 level of resources required to make change 3.3 complexity of the new approaches 3.4 team of willing change agents	*The degree to which an innovation is perceived as difficult to understand and use i.e., the perceived difficulty of implementing changes to dietetics education practice*
4.0 Trialability	4.1 pilot programs/others trying first 4.2 feedback mechanisms that allow educators to refine and improve new approaches 4.3 development of shared understanding or knowledge about innovation	*The degree to which an innovation can be experimented with before deciding to adopt it i.e., the opportunity to experiment with changes to dietetics education practice before fully committing*
5.0 Observability	5.1 ability to collect data on student outcomes 5.2 impact of new approach 5.3 share success stories with other educators 5.4 marketing tool/point of difference	*The degree to which the results of an innovation can be observed by others i.e. the degree to which the results of changing dietetics education practice can be observed and measured*
6.0 Communication channels	6.1 within universities/institution 6.2 across universities/institution	*Content exchange of a new idea. The means at which a new idea gets from one individual to another i.e., stories of success with concept-based curricula being transferred across universities*
7.0 Time	7.1 stage of change over time	*Time taken from learning about innovation to adopting the new practice.*
8.0 Social system	8.1 social structure 8.2 communication structure 8.3 norms 8.4 change agents	*The social structure in which the innovation is presented to– individuals, groups or organizations and their response to it and developing a community to progress the idea*

## Results

Twenty interviews with experienced dietetic educators were completed. Participants had an average of 14.3 years working in education (range 3 to 30 years) and 26 years as a practising dietitian (range 11 to 35 years). All states of Australia and one individual from New Zealand were represented (Qld = 5; Vic = 5; NSW = 3; WA = 4; NZ = 1; ACT = 1; SA = 1). Participants held curriculum development, disciplinary and course/program leadership positions.

### Research Question 1. What are the key factors influencing educational change in health professions education?

Three themes were identified that support understanding of how health professions educators may navigate proposed education change.

### Change champions

The role of education theory and evidence was seen as critical for change. This was described as being necessary to get other individuals to commit to change, and for deeper understanding of what is required for advocacy on the proposed approach to university and accreditation stakeholders. Many participants expressed their vulnerability in this area. They did not see themselves as the leader that would drive change despite their clearly articulated and defined role in program or curriculum leadership. Participants viewed this as something someone who was the expert in this area within their universities would lead, and then others would then follow.

*“So people…. who are taking this very [in-depth] approach to curriculum development and pedagogy, are vital to the profession, because we need people who can lift themselves out of the, you know, the minutia”* (participant #1).

Change champions were described to be experts in the education area who required a team of supportive staff to enable them to enact change. Teams of academic staff who were deeply committed to the objectives and worked effectively together as a team, were described as critical for success. While there was acknowledgement that change resistors are likely to be present, this can be overcome if the majority of the team were on board. A few participants described extraordinary education teams that they worked with which had enabled them to embrace a range of other curriculum transformations in the past, although many mentioned change fatigue after the COVID-19 pandemic. Leadership at a faculty and university level was also reported as important whereby the faculty or university structures, policies and systems allowed teams to introduce new curriculum. Change required leadership both to establish the vision, support the team to achieve this, and navigate the barriers placed by the university and other stakeholders along the way. A culture of growth mindset was needed.

*“I think innovation comes about when people are prepared to be brave, take a risk, not being afraid to stuff it up kind of thing.”* (participant #2).

Ground up or codesign approaches to engaging stakeholders from the beginning of the change process, in particular students and placement partners, were described as useful based on past experiences. The relationships between these parties needed to be fostered if change was to be a success. The few participants who had championed change in curriculum in the past were confident that they could embark of any change to curriculum and clearly articulated the change process required to do this.

*“There’s always resistance to change. People don’t like change, it’s uncomfortable. But, you know, change is inevitable”* (participant #3).

### Concerns

Fear drove some behaviour around curriculum transformation for participants. All participants described fear of accreditation, specifically accreditation review teams, not supporting innovations or difference, and therefore this forced education teams to ‘play it safe’ only making small changes.

*“if an accreditor didn’t understand that, we could be dragged over the coals” (*participant #6).

Some participants suggested that presenting the evidence of why they were changing and the impact of the approach to accreditation teams, lead to more confidence and resulted in accreditation bodies being supportive of anything new.

Hospital dietitians and clinical educators were feared by many participants due to the challenges of bringing them on board to any educational change. Dietitian stakeholders were highlighted as critical to the social system for which change in dietetics education occurred. The complex tapestry of communication between educators themselves, accreditation authorities, placement partners and relevant university stakeholders required to achieve social reform was explained. Participants expressed little confidence that they could talk through the benefits of concept-based approaches and convince practitioners in the field to change. This layer of complexity to navigate change in dietetics curricula was seen as a challenge for many.

*“clinical dietitians, jumping up and down and saying, oh, but you haven’t included paediatric, you know, heart disease. you know, some [rare] genetic condition.”* (participant #18).

Engagement of stakeholders was dominant in the participants’ talk. The need for stakeholder knowledge and attitudinal change to any education they hoped to incorporate, was described as critical to success. Key stakeholders were perceived to be students, academics, placement providers and the accrediting body. In addition, university and external governance parties, for example associate deans of education and accreditation councils were also key stakeholders. No participants talked of patients or consumers as stakeholders. Ensuring a high level of knowledge of a concept-based approach was described as critical for change together with changing philosophies and perspectives, especially as placement stakeholders were seen as holding traditional and historical views of what dietetics education needed to be. Listening to the perspectives of stakeholders and providing them with the knowledge and benefits of the concept-based approach might support them to manage the challenges they face was described as key.

Despite these challenges, participants clearly articulated the social structure of dietetics education and the communication required to achieve effective change. Over and above individual agents, organisations such as the accreditation body and the Council of Deans for nutrition and dietetics, together with informal social structures that facilitated conversations about evidence-based education practice, were clearly articulated as the mechanisms from which to communicate, create buy-in, and achieve change. Existing communities of practice in dietetic education were highlighted as mechanisms to manage these concerns.

*“if there was anything if anyone was willing to share, you know, you have like a community of practice, if people were willing to work together…. it’s very valuable.”* (participant #20).

### Complexity

Participants acknowledged the increasing pressure on curricula due to the changing landscape of the health system and workforce needs.

*“we can’t just keep adding things into the curriculum. You have to change the, what a curriculum looks like”* (participant #12).

They recognised they needed to consider other approaches to manage and navigate through this challenge. The complexity of the systems and structures in which university education takes place was viewed as challenging to navigate. Reports of cumbersome education governance, with changes to curriculum taking extensive time to move through the approval processes were dominant, although a few participants described supportive education governance systems that were enabling to innovation and change. For some participants that had undergraduate programs of study, the compatibility with existing curriculum and ability to impact change was described as impossible. This was due to the lack of control over some of the generic units of study, usually in biosciences, taught in their program. Those with post graduate programs saw greater potential and ability to control the approach to change as they owned or managed all content in the curriculum. The time taken for change to curriculum as part of these systems, together with the time required to bring teams together, was part of the complex system for which curriculum change occurs. Dietetics education practice was viewed as conservative and deeply rooted in historical practice, with few evidence-based innovations. This cultural complexity fuelled resistance to change.

*“They’ve been educated however long ago. And to some extent, a lot of them are frozen in that in that period, if they haven’t gone on and done extra education”.* (participant #7)

***Research Question 2. Can diffusion of innovation theory explain what would support or hinder change towards a concept-based approach to the education of future nutrition and dietetics professionals?***

**Fig. 1:**
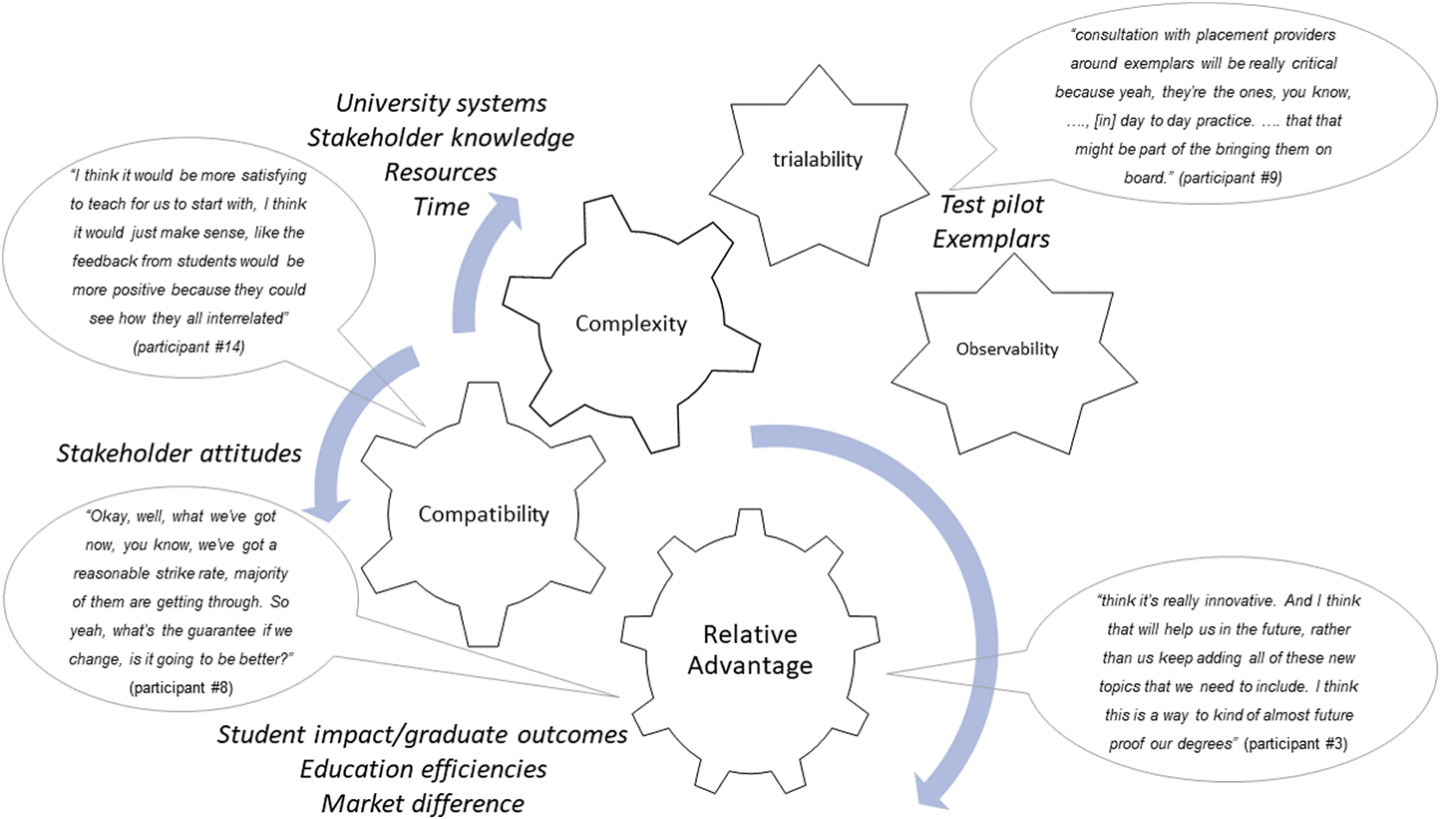
Illustration of how diffusion of innovation theory may explain change in dietetics education

The diffusion of innovation theory could explain how this change may occur emphasising key elements of this theory that are important in this context (Fig. 1).

Seeing the *relative advantage* of the new approach and that it would be better than the current state was evident. There was broad support for the idea of concept-based curricula approaches to manage the issue of overcrowding. Improved student outcomes and workforce preparation were the main drivers for change and advantage of a concept-based approach. Participants described not just changing for change sake, but that there needed to be impetus as the predicted outcomes for students were better than the current state. A concept-based curriculum that produced critical thinkers was viewed as a potential marketing strategy for universities to place individual courses above the rest.

Participants with a deep understanding of pedagogical theory could see the benefits of concept-based approaches and were not afraid of embarking on curriculum change. They articulated that the alignment to national competency standards for dietitians was a key strength supporting *compatibility* to existing curricula. Some perceived that this would require rethinking rather than major structural change to curricula, while others perceived that the change would conceptually be significant and therefore require more work. 

Resources and time dominated the talk regarding the potential *complexity* of the change. Existing academic workload models were a necessary consideration for resourcing and space for change. A team of academic staff willing and enabled to lead the change was required. Participants reported wanting a program to *trial* and *observe* the approach and share their learnings. There was a reluctance to be the first to try, given this was stated as something they did not have deep knowledge about. The need for exemplars was viewed as essential to the trialability of the approach. These exemplars were perceived as a useful way forward to manage emerging evidence of the changing nature and the future of the dietetics workforce.

## Discussion

Our study aimed to understand how Australian and New Zealand nutrition and dietetic educators may navigate proposed education change to core concepts in dietetic curricula and if theory could assist better understanding education change. We found that change champions with education expertise and leadership skills were essential influencers of change. Concerns and fears of accreditation and stakeholder perspectives, together with the challenges of navigating the complex system of health professions education, also influenced views on change. The diffusion of innovation theory explained the change towards a concept-based approach. While all elements of the theory were identified as critical to change, the need to be clear on the relative advantage of the new approach, see compatibility of the approach with current structures and systems, and have evidence from trialling and observing the new approach in action, were dominant in the participants’ narratives. Infiltrating the social system of dietetics education through existing communities of practice were reported as key.

Change in the culture of dietetic education is needed to allow innovation in curricula that prepares graduates for future practice (Ferguson et al., 2017). While leadership and scholarship have been recognised previously as key to changing culture and practice (Ferguson et al., 2017), our study further highlights the importance of leadership from those that have deeper understanding of evidence informed pedagogy. These findings together with existing literature, (Hay et al., 2022; McKimm & McLean, 2020; Sundberg et al., 2017) highlight that to change education practice and culture, we need to invest in future leaders. There is limited literature that provides direction on how to develop education leaders such that they are equipped to lead this change, although studies have suggested that leaders need to create legitimacy and expert status as educators in different ways (Sundberg et al., 2017). Our findings also highlighted that participants were fatigued with change. This was reported to be due to the timing of the study being at the tail-end of the COVID-19 global pandemic which in Australia and New Zealand caused extraordinary disruptions to health professions education, but may have also reflected fatigue at an institutional level. Yet change is inevitable in health professions education. Without strong quality improvement methods in health professions education, education quality and graduate outcomes will be compromised. If we are to truly embrace competency-based education, pivoting our education to meet population health and health system and service needs is required. Drawing on theories of change, as our findings have, may support educators and education systems to facilitate change.

In addition to activity theory (Law et al., 2022), and leadership (Velthuis et al., 2018), this study shows the value of diffusions of innovation in facilitating change more broadly in health professions education, and may also explain the resistance to curriculum reform specific to nutrition and dietetics. Diffusion of innovation theory effectively recognises the system in which health professions education operates, in particular how people within that system learn and make decisions about new practice. Using this theory as a tool to support innovators to consider what is required for change may be useful for leaders wishing to facilitate significant education change. This theory suggests that ensuring there is knowledge of the new innovation, and using persuasive techniques to gain support for the new practice, supports decisions to implement and continue the new practice. The ‘diffusion’ occurs when the system is different to what it was prior to the change. Given that it is clear that research evidence has little impact on education practice in health profession education (Ellaway et al., 2024), it is perhaps time to consider greater use of change theories in practice. Our study also highlights the important role of the communication and the social systems in which change occurs. Stories of success with concept-based curricula between individuals and across different universities is important. The role of educator communities of practice or networks may be a means to support ideas transferring between individuals and across education institutions and progress implementation. In line with communities of practice theory (Wenger-Trayner et al., 2014), learning involves meaningful participation with like-minded colleagues with a shared interest as they navigate new ideas. Motivating educators by learning together through shared projects and activities may support change. There is a need to further explore how informal and formed communities of practice can facilitate change in health professions education.

Our study focused on the context of dietetics education and faculty perspective in Australia and New Zealand. The transferability of our findings to other health professions is likely but unknown, as is the attitudes of others involved in dietetics education, for example, clinical educators, students, and administrators. The key finding that students and placement partners are stakeholders in facilitating change, provides guidance that future research should explore how they may engage with change towards concept-based approaches. The core concepts were developed for dietetic education internationally, however, it is recognised that other countries may have differing perspectives to those in Australia and New Zealand. Using theory to both code and interpret data provides strength to our analysis supporting translation more broadly.

## Conclusion

This study aimed to understand how nutrition and dietetics educators navigate proposed education change towards concept-based curriculum. Three main themes were identified from the interviews; the need for change champions, concerns about change, and the complexity of the education system. Diffusion of innovation theory helped to explain the resistance to change towards a concept-based approach to the education of future dietetics professionals and is relevant to health professions education more broadly. There was a recognised need for exemplars that were trialled by leaders to support change to concept-based curricula. Supportive mechanisms such as communities of practice and engagement with other stakeholders is required for future innovation and change.
